# Editorial: New insights and perspectives on traumatic brain injury: integration, translation and multidisciplinary approaches

**DOI:** 10.3389/fneur.2024.1427320

**Published:** 2024-06-24

**Authors:** Jian Shi, Zhou Zhou, Xianping Du, Maria Jose Cavagnaro, Jifeng Cai

**Affiliations:** ^1^Department of Critical Care Medicine and Hematology, The Third Xiangya Hospital, Central South University, Changsha, China; ^2^Division of Neuronic Engineering, KTH Royal Institute of Technology, Stockholm, Sweden; ^3^School of Marine Engineering and Technology, Sun Yat-sen University (Zhuhai Campus), Zhuhai, China; ^4^Department of Neurosurgery, School of Medicine, Stanford University, Palo Alto, CA, United States; ^5^FuRong Laboratory, Changsha, China; ^6^Department of Forensic Science, School of Basic Medical Science, Central South University, Changsha, China

**Keywords:** traumatic brain injury, clinical and translational neurology, mTBI, multidisciplinary science, clinical evaluation, real world research, forensic authentication, artificial intelligence

## Introduction

Traumatic brain injury (TBI) remains one of healthcare's most significant challenges and policy making. TBI could not only lead to long-term functional impairment but also a decrease in quality of life. Lowering the mortality risk and benefitting survivors' living quality remains the target of neurotrauma studies globally. With the advent of novel TBI-related clinical and basic research approaches, diagnostics, therapeutics research, and novel and multidisciplinary methods have emerged. Despite promising progress, no completely effective treatment prevents or minimizes TBI and its related neurological and psychiatric sequelae. Understanding the mechanisms underlying the pathophysiology, treatment progress, and clinically based translational and engineering research on TBI may pave the way for potential treatment targets, diagnostic markers, and preventive methods that ultimately lead to efficacious therapeutic strategies. In this Research Topic, we are delighted to have received several outstanding studies focusing on various aspects of TBI-related research, which have provided us with numerous insights and inspirations. We will provide a detailed review based on the collection status of this Research Topic and the development of the field.

## Perspectives

The 12 diverse research articles published on this Research Topic not only make significant contributions to integration, translation, and multidisciplinary aspects but also essentially cover important references ranging from prevention, personalized stratification, precision treatments to prognosis. This aligns with the viewpoints proposed by The Lancet Neurology Commission on TBI (1), mutually corroborating and highlighting the significance and value of this Research Topic. They specifically include and cover the following three main aspects.

### Basic and translational research

This Research Topic, focusing on basic and translational research, delves into research articles and reviews on TBI-related mechanisms and experimental methods, including the following articles: *Diffusion basis spectrum imaging detects subclinical traumatic optic neuropathy in a closed-head impact mouse model of traumatic brain injury* (Yang et al.) and *Models of traumatic brain injury-highlights and drawbacks* (Zhao et al.).

They articulate various aspects of TBI models from different perspectives. As we know, TBI is a combination of anatomical and functional damage to the brain following direct mechanical injury. The brain damage caused by TBI is a mixture of structural, cellular, and vascular damage. The structural damage caused by the initial impact activates complex molecular and cellular cascade reactions. Therefore, animal models commonly used for TBI research include fluid percussion injury (FPI), controlled cortical impact injury (CCI), weight drop impact acceleration injury (WDIAI), and blast injury models, each with their own characteristics and limitations. Further improvement and optimization of these models require more excellent research to support and corroborate (2, 3) (Zhao et al.).

In another aspect of basic research, we have also received two sets of examples from bibliometrics and scientometrics which are: *The bibliometric and altmetric analysis of chronic traumatic encephalopathy research: how great is the impact?* (Guan et al.) and *An exhaustive analysis of post-traumatic brain injury dementia using bibliometric methodologies* (Sang et al.). Scientometrics and bibliometrics are methodological approaches in which the scientific literature itself becomes the subject of analysis. There has already been a series of concentrated applications of it in clinical disease-related research (4–7). Scientometrics and bibliometrics often involve monitoring research, assessing the scientific contributions of authors, journals, or specific works, as well as analyzing the dissemination process of scientific knowledge. Additionally, several excellent reviews included in the Research Topic have provided us with new insights from multiple dimensions, including: *Advances in understanding the pathogenesis of post-traumatic epilepsy: a literature review* (Fang et al.), *Post-traumatic olfactory dysfunction: a scoping review of assessment and rehabilitation approaches* (De Luca et al.), and *History of concussion and lowered heart rate variability at rest beyond symptom recovery: a systematic review and meta-analysis* (Wesolowski et al.). These detailed and in-depth summaries and descriptions provide valuable references for us to gain a deeper understanding of TBI.

### Clinical research

In this Research Topic, research articles and reviews that delve into in-depth exploration from the perspective of clinical practical issues include: *The prognostic value of an age-adjusted BIG score in adult patients with traumatic brain injury* (Bai et al.), *Chronic kidney disease as a predictive factor for poor prognosis in traumatic brain injury among older adults: a case-control study* (Mo et al.), and *Epidemiological characteristics for patients with traumatic brain injury and the nomogram model for poor prognosis: an 18-year hospital-based study* (Guo et al.) with *Secondary hyperperfusion injury following surgical evacuation for acute isolated epidural hematoma with concurrent cerebral herniation* (Huang et al.). Each one is an exciting clinical-relevant study, presenting not only the current panorama and hotspots of clinical exploration in TBI comprehensively, but also providing important research references for diagnosis, treatment, and potential applications, especially in all aspects closely related to TBI prognosis. To enrich TBI-related clinical research, methods such as expanding the sample size, increasing the number of study subjects, conducting multicenter collaborative research, strengthening clinical trials and translational research, establishing comprehensive data collection and analysis systems, and conducting real-world studies have all been increasingly utilized and have yielded beneficial research outcomes (8–13).

### Forensic and precision identification research

In the field of TBI research, another important area is forensic and precision identification research, which intersects closely with disciplines such as early biomarker discovery, functional assessment, and imaging. In this domain, the identification of mild traumatic brain injury (mTBI) is particularly crucial. mTBI often presents with mild clinical symptoms and subtle imaging findings, making it prone to misdiagnosis or underdiagnosis, especially when the severity of injury does not necessarily correlate with the degree of post-injury functional impairment. To address these challenges in forensic clinical and judicial practice, numerous scholars have provided important research directions, including exploration of biomarkers, studies on rapid detection methods, in-depth exploration of medical imaging, and interdisciplinary research based on deep learning artificial intelligence (14–21). Including the research article *Translational medical bioengineering research of traumatic brain injury among Chinese and American pedestrians caused by vehicle collision based on human body finite element modeling* (Yan et al.), in this Research Topic, It is also an important interdisciplinary translational study with relevance to forensic identification. In this research domain, there is a need to explore more early potential biomarkers of mTBI, develop sensitive and rapid multimodal identification methods, and ultimately provide a solid theoretical foundation and practical basis for the resolution of forensic precision identification issues.

## Conclusion and final considerations

The goal of this Research Topic is to disseminate high-quality research on traumatic brain injury (TBI) and related fields, with a particular focus on comprehensive and in-depth studies involving integration, translation, and multidisciplinary approaches. The aim is to bridge the gap between basic neuroscience knowledge and the unmet needs in brain health through these methodologies. To achieve this, the Research Topic presents a collection of compelling research studies and reviews that showcase advancements in neuroscience and explore multidisciplinary and interdisciplinary integration. We hope that this Research Topic will contribute to advancing the understanding of this complex and multifaceted field. However, beyond the research content and insights provided by this Research Topic, we must also acknowledge that the complexity of different perspectives often results in the omission of the service provision system, which encompasses prevention, pre-hospital and hospital care, rehabilitation, and resocialization of the disabled. This omission is closely related to the role of public health and the quality of services provided in accordance with evidence-based medicine. Addressing these aspects would enhance the impact of the contribution by mobilizing the professional community in support of TBI victims. Therefore, additional aspects should be considered. These include developing comprehensive strategies for the prevention of TBI, improving pre-hospital care protocols to ensure timely and effective initial treatment, enhancing hospital care with cutting-edge medical practices, and establishing robust rehabilitation programs that focus on both physical and cognitive recovery. Additionally, creating supportive environments for the resocialization of disabled individuals is crucial. By incorporating these elements, we can create a holistic approach that not only advances scientific understanding but also significantly improves patient outcomes and quality of life for TBI sufferers.

We hope that this Research Topic will contribute to advancing the understanding of this complex and multifaceted field. We extend our heartfelt gratitude to the various groups that submitted their scientific findings to this Research Topic, as well as to the reviewers who generously dedicated their time, effort, and expertise to enhance the quality of each study.

## Author contributions

JS: Writing – original draft, Writing – review & editing. ZZ: Writing – review & editing. XD: Writing – original draft, Writing – review & editing. MC: Writing – review & editing. JC: Writing – original draft, Supervision.
